# A Unique Cause of Intestinal and Splenic Infarction in a Sickle Cell Trait Patient

**DOI:** 10.1155/2013/580453

**Published:** 2013-05-07

**Authors:** Sofya H. Asfaw, Gavin A. Falk, Gareth Morris-Stiff, Ralph J. Tuthill, Matthew L. Moorman, Michael A. Samotowka

**Affiliations:** ^1^Department of General Surgery, Digestive Disease Institute, Cleveland Clinic Foundation, 9500 Euclid Avenue, Cleveland, OH 44195, USA; ^2^Department of Anatomic Pathology, Pathology and Laboratory Medicine Institute, Cleveland Clinic Foundation, 9500 Euclid Avenue, Cleveland, OH 44195, USA

## Abstract

Sickle-cell trait is a common genetic abnormality in the African American population. A sickle-cell crisis in a patient with sickle-cell trait is uncommon at best. Abdominal painful crises are typical of patients with sickle cell anemia. The treatment for an abdominal painful crisis is usually medical and rarely surgical. We present the case of a cocaine-induced sickle-cell crisis in a sickle-cell trait patient that resulted in splenic, intestinal, and cerebral infarctions and multisystem organ failure necessitating a splenectomy, subtotal colectomy, and small bowel resection. This case highlights the diagnostic dilemma that abdominal pain can present in the sickle-cell population and illustrates the importance of recognizing the potential for traditionally medically managed illnesses to become surgical emergencies.

## 1. Introduction

Sickle-cell anemia (SCA), an autosomal recessive disease, results from a valine for glutamic acid substitution at position six of the *β*-globin gene of hemoglobin (Hb). When the sickle hemoglobin (HbS) molecule is deoxygenated, there is a hydrophobic interaction between this and other hemoglobin molecules that trigger an aggregation into large polymers resulting in sickle-shaped deformities of the red blood cell (RBC). When RBCs sickle, the common critical manifestations are vasoocclusive, sequestration, hemolytic, and aplastic crises [1]. These sequelae almost always occur in individuals homozygous (HbSS) for the mutation that causes SCA. Sickle-cell trait (SCT) is the heterozygous form of the disease (HbAS), and people with this genotype rarely exhibit disease manifestations. According to the Centers for Disease Control and Prevention, SCA occurs in about 1 in 500 African American births while the trait occurs much more frequently with an incidence of 1 in 12 African American births.

Cocaine, a central nervous system stimulant and commonly used drug of abuse, is known to cause vascular ischemia through a multifactorial mechanism. We present a rare case of a cocaine-induced sickle-cell crisis in a patient with SCT leading to splenic and intestinal infarction.

## 2. Case Report

A 50-year-old female with SCT presented to the emergency department of our institution with a three-day history of diffuse abdominal pain, emesis, and diarrhea. On questioning, she admitted to recent cocaine use, and blood tests showed a serum total bilirubin of 2.5 mg/dL (normal: 0.3–1.9 mg/dL). 

**Figure 1:**
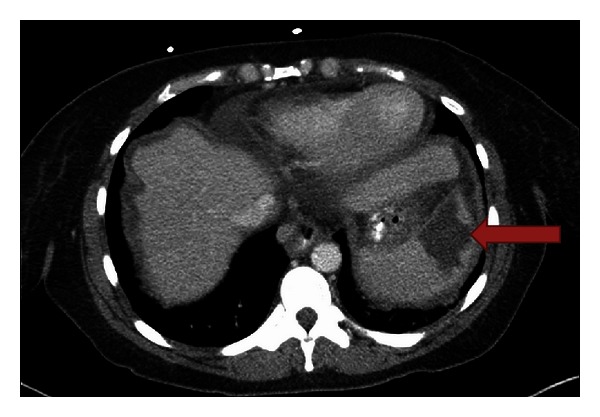
Axial CT scan showing a wedge shaped low-attenuation area in the spleen representing an area of infarction (red arrow).

Physical examination revealed a distended and diffusely tender abdomen. She had a blood pressure of 124/77, pulse rate of 93, temperature of 37.3°C, and an oxygen saturation of 95% on room air. A computed tomography (CT) scan was performed and showed a splenic infarct, mildly dilated small bowel loops, and moderate ascites (Figure 1).

After 48 hours of improving symptoms and signs, on hospital day 3, the patient complained of increasing abdominal pain and suddenly became hemodynamically unstable, requiring endotracheal intubation and initiation of vasopressor support. On exam, she was diaphoretic and minimally responsive, her blood pressure fell to 69/56, and her heart rate elevated to 115. Laboratory investigations revealed a severe lactic acidosis and a serum total bilirubin of 9.5 mg/dL. The patient was brought to the operating room, and an exploratory laparotomy was performed. Operative findings were a diffusely ischemic appearing colon, infarcted spleen, and necrotic omentum. A splenectomy, omentectomy, subtotal colectomy, and small bowel resection were carried out.

Postoperatively, she developed multisystem organ failure including acute renal failure requiring continuous veno-venous hemodialysis, ischemic hepatitis (“shock liver”), cardiopulmonary failure, and bilateral anterior and middle cerebral artery territory infarcts, with prominent mass effect from bilateral internal carotid artery occlusion by sickled cells. After discussion with her family, supportive care was withdrawn. 

**Figure 2:**
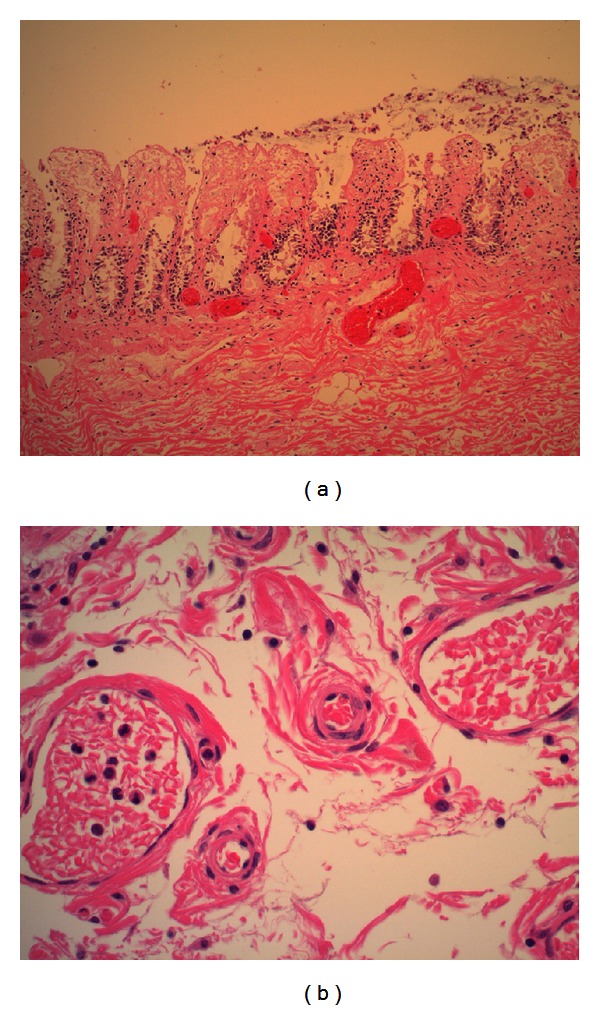
(a) shows colon with congestion of capillary venules and ischemic necrosis of overlying mucosa. (b) shows sickle-shaped red blood cells in congested capillary venules.

Final pathology showed vascular congestion with sickled RBCs throughout her omentum, spleen, colon, and terminal ileum consistent with sickle crisis. Changes of pseudomembranous colitis were not identified. Tissue gram stain was negative for bacteria, and PAS stain after diastase was negative for fungi (Figure 2).

## 3. Discussion

While splenic infarction is more commonly seen in patients with SCA, a handful of cases have been reported describing splenic infarcts in SCT patients. Most cases have been associated with high-altitude flying [2–6] as at elevated altitudes; the alveolar partial pressure of oxygen (pO2) can drop significantly. As oxygen saturation decreases, there is an increase in polymerization and deformability of the hemoglobin S molecule that results in sickling [7].

Gastrointestinal manifestations such as ischemic colitis have been reported with equal rarity [8–10]. It has been postulated that the gastrointestinal tract has a rich collateral blood supply that may protect the bowel from the effects of static sickled RBCs [11]. The bowel is less susceptible to ischemia because of the lower degree of the oxygen extracted (15–20% of oxygen delivered) and arteriovenous shunting in the bowel wall [12]. Abdominal pain may often accompany sickle-cell crisis, which can make it difficult to distinguish from other causes of an acute surgical abdomen. The incidence of abdominal pain in sickle-cell crisis is reported to be from 30 to 57% [13]. When vasoocclusion occurs in the mesenteric, hepatic, or pulmonary distributions, it is termed “girdle syndrome” as it results in pain in a girdle-like distribution [13]. Typically, pain resolves with conservative therapy, but occasionally surgical intervention may be warranted. 

Cocaine use is associated with an increased circulating concentration of catecholamines. The oxidative metabolism of catecholamines may have a damaging effect on the heart muscle and other organs. It is hypothesized that cocaine-induced vascular ischemia, obstruction from small vessel vasospasm, vasoconstriction, or thrombosis may lead to infarction. Cocaine-associated infarctions in people without SCA or SCT have been reported in various organs including the skin, aorta, intestines, and spleen [14].

There are few reports in the literature that describe the impact of cocaine use in sickle-cell patients. There is one case series which reports cocaine abuse resulting in acute painful episodes and multisystem organ failure in SCA [15] and another report of a cocaine-associated death in an SCA patient [16]. To our knowledge, there are no reports of cocaine-associated sickle-cell crisis and its sequelae in patients with SCT. We propose that cocaine-induced vasoconstriction contributed to a cyclical cascade of tissue hypoxia, RBC sickling, and vasoocclusion which led to splenic, intestinal, omental, and cerebral infarctions and acute multisystem organ failure. Cocaine use in sickle-cell patients may elicit a sickle-cell crisis leading to a deadly cascade of events. 

While abdominal pain may mark a crisis in the SCA population, it is not commonplace in SCT patients. Consequently, a patient with abdominal pain can present a diagnostic dilemma in either of these populations, but more so in the SCT patient. An abdominal painful crisis may be indistinguishable from other intra-abdominal pathology and is usually self-limiting. However, it is important that clinicians are aware that patients with SCT can suffer from sickle-cell crises, know the risk factors associated with its development, and can recognize the signs and symptoms of its presentation.

A comprehensive history, physical examination, and consideration of a sickle crisis in the differential diagnosis of an SCT patient presenting with abdominal pain are vital. This can result in its early diagnosis and timely initiation of appropriate management, including intensive supportive care, which may obviate the need for surgical intervention and prevent the development of a potentially irreversible cascade of events.
